# DeepKla: An attention mechanism‐based deep neural network for protein lysine lactylation site prediction

**DOI:** 10.1002/imt2.11

**Published:** 2022-03-15

**Authors:** Hao Lv, Fu‐Ying Dao, Hao Lin

**Affiliations:** ^1^ Key Laboratory for Neuro‐Information of Ministry of Education, School of Life Science and Technology, Center for Informational Biology University of Electronic Science and Technology of China Chengdu Sichuan China; ^2^ Department of Molecular Life Sciences University of Zurich Zurich Switzerland; ^3^ School of Biological Sciences Nanyang Technological University Singapore Singapore

**Keywords:** attention mechanism, bidirectional gated recurrent units, convolutional neural network, embedding layer, lactylation

## Abstract

As a newly discovered protein posttranslational modification, lysine lactylation (Kla) plays a pivotal role in various cellular processes. High throughput mass spectrometry is the primary approach for the detection of Kla sites. However, experimental approaches for identifying Kla sites are often time‐consuming and labor‐intensive when compared to computational methods. Therefore, it is desirable to develop a powerful tool for identifying Kla sites. For this purpose, we presented the first computational framework termed as DeepKla for Kla sites prediction in rice by combining supervised embedding layer, convolutional neural network, bidirectional gated recurrent units, and attention mechanism layer. Comprehensive experiment results demonstrated the excellent predictive power and robustness of DeepKla. Based on the proposed model, a web‐server called DeepKla was established and is freely accessible at http://lin-group.cn/server/DeepKla. The source code of DeepKla is freely available at the repository https://github.com/linDing-group/DeepKla.

## Highlights

- We presented the first computational tool, termed DeepKla, to identify Kla sites in rice.
- Supervized embedding layer, convolutional neural network, bidirectional gated recurrent units, and attention mechanism layer were applied to train the model.
- A robust, generalized, and convenient web‐server of DeepKla was established at http://lin-group.cn/server/DeepKla.

## Graphical abstract

We presented the first computational tool, termed DeepKla, to identify Kla sites in rice. Supervised embedding layer, convolutional neural network, bidirectional gated recurrent units, and attention mechanism layer were applied to train the model. A robust, generalized, and convenient web‐server of DeepKla was established at http://lin-group.cn/server/DeepKla.

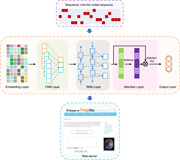

## INTRODUCTION

Lysine lactylation (Kla) is a new type of posttranslational modification (PTM) that exists in mammalian, plant, and fungi cells [1–3]. Biochemically, Kla introduces a small lactyl group on the *ε* amine group of the lysine residue, with a mass of 72.021 Da [4]. Accumulating evidence indicates that lactylation is associated with inflammatory response [1, 5], progression of lung fibrosis [6], and cellular reprogramming [7]. However, the regulatory role of Kla in influencing the establishment of cellular processes is still unclear.

The conventional characterization of Kla sites is a mass shift‐based high‐performance liquid chromatography‐tandem mass spectrometry (MS/MS) technique following peptide synthesis and isotopic [4]. However, the drawbacks of experimental methods preclude the proteome‐wide identification of Kla sites. Thus, there is a need for computational methods to fill in the experimental void.

To the best of our knowledge, there is no computational model for Kla sites identification in rice. Thus, in this study, we proposed a novel deep learning‐based model, named DeepKla, to accurately identify protein lactylation sites. As an integrated deep learning architecture, DeepKla consists of four closely connected sub‐networks including a word embedding layer, convolutional neural network (CNN), bidirectional gated recurrent units (BiGRU), and attention mechanism layer. Specifically, the embedding layer automatically extracted sequence features using protein sequences as the only input, thereby avoiding the biased features resulting from artificially designed. In addition, BiGRU and the attention mechanism were used to capture long‐range and key position information from protein sequences, respectively. Benchmarking experimental results demonstrated that the robust representations generated by the embedding layer and CNN–BiGRU‐attention mechanism layer have a strong predictive performance in identifying Kla sites. We believe that the proposed architecture can also address other PTM sites identification problems better than previous methods.

## METHODS

### Benchmark data set

In this study, lactylation data for rice were collected from literature as training data [2]. The annotated lactylation sites on lysine (K) were used as positive data, while the same amino acid excluding annotated lactylation sites from the same proteins was regarded as the negative data. According to the preliminary evaluation using windows of different lengths, a window size was set to 51 to maximize the extraction of Kla site information. To construct a nonredundant benchmark data set, the CD‐HIT program [8] was used with the sequence similarity threshold of 30%. As a result, many negative samples were yielded. To balance the positive and negative data, we used oversampling of positive samples to keep the positive and negative data with a ratio of 1:1. In addition, we collected 273 Kla data in *Botrytis cinerea* as testing data from the literature [3] to objectively evaluate the proposed model. A detailed description of the data has been listed in Table 1.

**Table 1:** The training data and independent data used in this study

Data type	Training data		Testing data	
	Positive	Negative	Positive	Negative
Number	1720	1767	177	177

### Sequence representation and architecture of DeepKla

**Figure 1:**
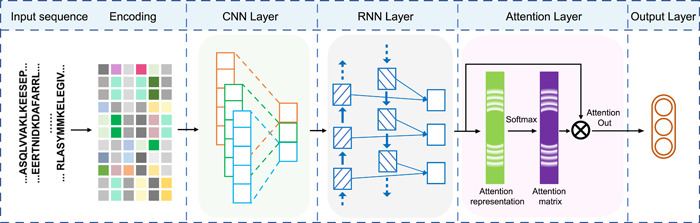
Flowchart of DeepKla. CNN, convolutional neural network; RNN, recurrent neural network

Figure 1 summarized our deep‐learning framework for Kla site prediction. Given a protein sequence, we coded it by a supervised embedding layer that has been successfully adopted in PTM site prediction [9, 10]. In the deep‐learning architecture of DeepKla, the multilayer CNN encoded an input protein sequence into a fixed two‐dimensional hidden state. Then, the two‐dimensional hidden state was fed into BiGRU. In addition to BiGRU architecture, the attention mechanism layer was also employed to capture the position information of protein sequences. The detailed descriptions of sequence representation and algorithm architecture design in Supporting Information.

The model was implemented in Keras (version 2.0.6) and carried out on a MacOS with 1.4 GHz Intel Quad‐Core i5. We chose the default learning rate for the RMSProp optimizer during the training process and used 64 as the batch size. Five‐fold cross‐validation was performed to determine the model structure and hyperparameters on the training data. To avoid overfitting, we selected three data points on the validation set for early stop.

## RESULTS

### Workflow of DeepKla

The entire workflow of DeepKla is illustrated in Figure 1. After the data collection and preprocessing, DeepKla assigns integers to each protein sequence. The CNN–BiGRU‐attention mechanism layers are performed iteratively to capture features of protein sequences. In the output step, a fully connected layer and a softmax layer are used to produce the result.

### Evaluate the performance

**Figure 2:**
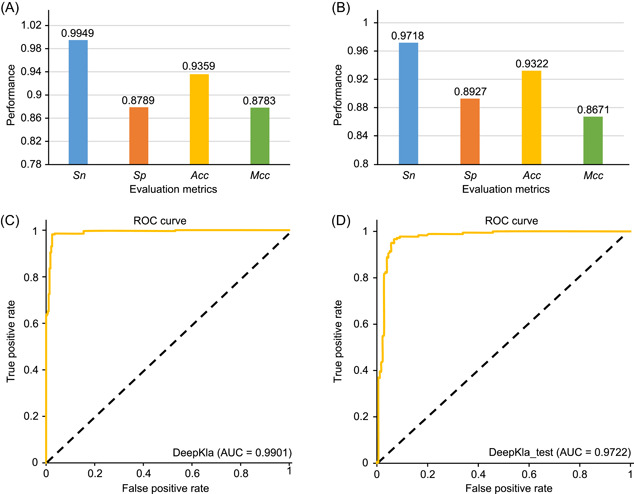
Illustration to show the prediction indexes for identifying Kla sites by using the training data set and independent data set. (A, B) The prediction indexes of training data set (A) and independent data set (B), respectively. (C, D). ROC curves of training data set and independent data set, respectively. The performance evaluation metrics are defined as *Sn* (sensitivity) = TP/(TP + FN), *Sp* (specificity) = TN/(TN + FP), *Acc* (accuracy) = (Sn + Sp)/2, *MCC* (Matthews correlation coefficient) = (TP × TN – FP × FN)/√ (TP + FP) (TP + FN) (TN + FP) (TN + FN), where TP = true positive, FP = false positive, TN = true negative, and FN = false negative. AUC, area under the ROC curve; ROC, receiver operating characteristic

To evaluate the prediction performance of DeepKla, five‐fold cross‐validation was performed. To do so, we randomly divided the training data set into five nonoverlapping subsets. In each validation step, four‐fifths of the data were used to train the model, whereas the remaining one‐fifth of the data were adopted to test its performance. The sensitivity (*Sn*), specificity (*Sp*), accuracy (*Acc*), Matthews correlation coefficient (*MCC*), and average receiver operating characteristic (ROC) of the five tests were plotted in Figure 2A,C. By taking different thresholds according to the scores by ROC curves, the area under the ROC curves (AUC) was calculated. It shows that the DeepKla could produce an AUC of 0.9901 (Figure 2C), demonstrating the robustness of DeepKla in identifying Kla and non‐Kla sites.

Independent data set has been widely used to assess the robustness of a specified classifier. Thus, we collected 273 Kla data in *B. cinerea* from the literature [3] to further evaluate the performance of DeepKla. After the same processing criteria as the benchmark data set, 177 Kla sequences and 177 non‐Kla sequences were obtained and displayed in Table 1. As shown in Figure 2B,D, we noticed that the DeepKla produced consistently satisfactory performance on *Sn* (0.9718), *Sp* (0.8927), *Acc* (0.9322), *MCC* (0.8671), and AUC (0.9722). This result indicated that DeepKla has excellent prediction ability and transferability to identify Kla sites.

We evaluated the contribution of different strategies that affect the performance of DeepKla. We compared the performance of DeepKla with and without attention mechanism layer by testing on the same independent data set. Results show that attention‐contained architecture obtained the better performance (accuracy = 94.07% [333/354]), while no attention architecture got the second‐best result (accuracy = 92.09% [326/354]). This result highlights the effectiveness of the attention mechanism that could capture key information in the Kla prediction problem. We also compared the performance of DeepKla under CNN–BiGRU‐attention mechanism and CNN–BiLSTM‐attention mechanism, respectively. The results showed that the architecture including BiLSTM produced weaker performance (accuracy = 85.59% [303/354]), indicating that the improved version of BiLSTM, that is, BiGRU, has more advantages in improving the prediction ability of Kla sites.

### DeepKla web server

For the convenience of peers, we built an online web server. The web server only accepts protein sequences in FASTA format. The server sets two input options, one is to directly paste the sequence to be predicted to the blank box, and the other is to upload the local folder when the number of query sequences is large. It should be noted that the query sequence cannot contain special characters such as “X,” otherwise the model will not recognize it and return an error report. After the job is finished, the prediction results are displayed in another interface, where all predicted Kla sites are visualized together with their probabilities.

## CONCLUSION

Here, we present DeepKla, an easily used and publicly available deep learning‐based tool for predicting Kla sites in rice. We use an embedding layer following a CNN–BiGRU‐attention mechanism layer to encode and learn representations of protein sequences. Comprehensive tests showed the robustness of DeepKla. We believe that our study will facilitate accurately predict the Kla sites with massive data.

## CONFLICTS OF INTEREST

The authors declare that there are no conflicts of interest.

## AUTHOR CONTRIBUTIONS

**Hao Lv**: coding, writing – original draft, conceptualization, writing – original draft. **Fu‐Ying Dao**: writing – original draft. **Hao Lin**: investigation, writing – review and editing, funding acquisition.

## Supporting information

Supporting information.

Supporting information.

## Data Availability

The authors provide the Python source code and benchmark data set of DeepKla model training and testing, which are freely available at https://github.com/linDing-group/DeepKla or http://lin-group.cn/server/DeepKla. Supporting Information (figures, tables, scripts, graphical abstract, slides, videos, Chinese translated version and update materials) may be found in the online DOI or iMeta Science http://www.imeta.science/.
