# Supplementary material for: DeepKla: An attention mechanism‐based deep neural network for protein lysine lactylation site prediction
Source: Imeta. 2022 Mar 15;1(1):e11. doi: 10.1002/imt2.11 (PMC10989745; doi:10.1002/imt2.11)
Supplement: Supplementary file 1 — Supporting information. [file IMT2-1-e11-s001.docx]

**DeepKla: an attention mechanism-based deep neural network for protein lysine lactylation site prediction**

Hao Lv1^,2^, Fu-Ying Dao1^,3^, Hao Lin^1^*

^1^School of Life Science and Technology, Center for Informational Biology, University of Electronic Science and Technology of China, Chengdu 610054, China.

^2^Department of Molecular Life Sciences, University of Zurich, Winterthurerstrasse 190, 8057 Zurich, Switzerland.

^3^School of Biological Sciences, Nanyang Technological University, Singapore 639798, Singapore

***CORRESPONDENCE:**

Hao Lin: [hlin@uestc.edu.cn](mailto:hlin@uestc.edu.cn)

**1. Sequence representation**

The essence of embedding layer in Keras [1] is a fully connected neural network, which turn positive integers (indexes) into dense vectors of fixed size. For a given protein sequence, a fixed-length digital vector was generated by replacing the amino acids with their corresponding encoders. If the length is less than “*max_length*”, we used function “*pad_sequence*” to amplify the length of protein sequence to 200 aa. By doing this, a protein sequence is converted to a sparse vector with many zeros. However, this ordinary encoding scheme cannot reflect the relationship between protein residues and their sequential and spatial neighbors. Thus, we used embedding layer to map amino acids to dense vectors by simulating protein sequences as documents and amino acids as words [2]. The semantic similarity between two arbitrary amino acids learned from large-scale sequences allows us to use the continuous metric notions of similarity to assess the semantic quality of individual amino acids. Embedding an amino acid can be done by multiplying the one-hot vector from the left with a weight matrix $W\in R^{d\times\left| V \right|}$, where $\left| V \right|$ is the number of unique amino acids and $d$ is the embedding size. Supporting that $v_{i}$ is the one-hot vector of an amino acid $x_{i}$ in a given protein sequence $x=x_{1}x_{2}\cdots x_{n}$, the embedding of $x_{i}$ can be represented as follows:

$e_{i}=Wv_{i},$ (1)

The weight matrix is randomly initialized and updated in a back-propagation fashion. After the embedding layer, an input sequence can be presented by a dense matrix $E_{d\times n}=\left( e_{1},e_{2}\cdots,e_{n} \right)$.

**2. Algorithm architecture design**

Here, we presented a hybrid deep-learning architecture consisted of CNNs, BiGRU, and attention mechanism layers, where CNNs were used to extract high-level motif features, BiGRU and the attention mechanism were used to capture long-range and key position information from protein sequences, respectively. The details of the architecture are as follows:

(1) Convolutional layer: The convolutional layer is a major building block of CNN, which contains a set of learnable filters where each filter is convolved with the input of the layer to encode the local knowledge of the small receptive field. This process helps conserve the dimensional relationship between numeric values in the vectors [3]. Thus, a 1D convolutional layer was used to construct a convolution kernel and then derive features encoded in the embedding layer [4].

(2) Rectified Linear Unit (ReLU): An additional non-linear operation was presented after every convolution operation. It aims to introduce the property of non-linearity into the model and produce a more desirable output. The output function of ReLU is as follows:

$f\left( x \right)=\max\left( 0,x \right),$ (6)

where $x$ is the number of inputs in a neural network.

(3) Pooling layer: Max pooling is a sample-based discretization process. It was used to down-sample the hidden-layer output matrix, reducing its dimensionality and allowing for assumptions to be made about features contained in the sub-regions binned. In this step, we set Max pooling stride equal to 2.

(4) Dropout layer: A technique which probabilistically dropping out nodes in the network for reducing overfitting and improving the generalization of deep neural networks. In this step, we set the dropout size equal to 0.25.

(5) BiGRU layer: GRU is a variant of LSTM and consists of a forget gate and an input gate. Compared with LSTM, GRU has simpler structure, fewer parameters, and better model convergence [5]. Suppose the input sequence is $\left\{ x1,x2,\cdots xn \right\}$, using GRU, we have $h_{t}^{1}={GRU}^{1}\left( x1,h_{t-1}^{1} \right)$. GRU is defined as follows:

$$\left\{ \begin{aligned} z_{t}=\sigma\left( W_{z}\left[ x_{t},h_{t-1}^{1} \right] \right) \\ r_{i}=\sigma(W_{r}[x_{t},h_{t-1}^{1}]) \\ \tilde{h}_{t}^{1}=\tanh(W_{h}[x_{t},r_{t}\times h_{t-1}^{1}]) \\ h_{t}^{1}=\left( 1-z_{t} \right)\times h_{t-1}^{1}+z_{t}\times\tilde{h}_{t}^{1} \end{aligned} \right.$$

BiGRU [6] consists of a forward GRU and a backward GRU, which can obtain sequential information of text from two perspectives.

(6) Attention mechanism layer： The core of the attention mechanism is to calculate dynamic adaptive weights based on probability distribution. The weight score can be calculated by follows:

$$e_{i}=x_{a}^{T}\tanh(w_{a}h_{i}+b)$$

Where $h_{i}$ is the hidden layer output, $w_{a}$ is the random initialization weight matrix, $x_{a}$ is the random initialization vector, and $b$ is the offset vector. Next, the weight score can be represented by:

$$\vartheta=\frac{exp(e_{i})}{\sum_{k=1}^{L} exp(e_{i}k)}$$

and the output vector $c_{i}$ weighted by the dynamic adaptive weight is:

$$c_{i}=\sum_{j=1}^{L} \vartheta\cdot h_{j}$$

(7) Dense layer: A neural network layer that is connected deeply, which means each neuron in the dense layer receives input from all neurons of its previous layer. Our task is to train a binary classification model to distinguish phosphorylation sites and non-phosphorylation sites. Therefore, in this step, we set the number of nodes equal to 2.

**REFERENCES**

[1] François Chollet. 2018. Keras: The python deep learning library. ***Astrophysics source code library*** ascl: 1806.1022.

[2] Hang Li, Xiu-Jun Gong, Hua Yu, Chang Zhou. 2018. Deep neural network based predictions of protein interactions using primary sequences. ***Molecules*** 23: 1923. <https://doi.org/10.3390/molecules23081923>

[3] Karim Abbasi, Parvin Razzaghi, Antti Poso, Massoud Amanlou, Jahan B Ghasemi, Ali Masoudi-Nejad. 2020. DeepCDA: deep cross-domain compound–protein affinity prediction through LSTM and convolutional neural networks. ***Bioinformatics*** 36: 4633-4642. <https://doi.org/10.1093/bioinformatics/btaa544>

[4] Nguyen Quoc Khanh Le, Edward Kien Yee Yapp, N Nagasundaram, Hui-Yuan Yeh. 2019. Classifying promoters by interpreting the hidden information of DNA sequences via deep learning and combination of continuous FastText N-grams. ***Frontiers in Bioengineering Biotechnology*** 305. <https://doi.org/10.3389/fbioe.2019.00305>

[5] Kyunghyun Cho, Bart Van Merriënboer, Caglar Gulcehre, Dzmitry Bahdanau, Fethi Bougares, Holger Schwenk*, et al.* 2014. Learning phrase representations using RNN encoder-decoder for statistical machine translation. ***arXiv*** <https://doi.org/arXiv:1406.1078>

[6] Caiming Xiong, Stephen Merity, Richard Socher.2016. Dynamic memory networks for visual and textual question answering. ***International conference on machine learning***:2397-2406.
