# Supplementary material for: DeepKla: An attention mechanism‐based deep neural network for protein lysine lactylation site prediction
Source: Imeta. 2022 Mar 15;1(1):e11. doi: 10.1002/imt2.11 (PMC10989745; doi:10.1002/imt2.11)
Supplement: Supplementary file 2 — Supporting information. [file IMT2-1-e11-s002.docx]

**Supporting Information**

Supporting information contains detailed descriptions of sequence representation and algorithm architecture design.
